# The Role of Occupational Therapy in Managing Food Selectivity of Children with Autism Spectrum Disorder: A Scoping Review

**DOI:** 10.3390/children8111024

**Published:** 2021-11-07

**Authors:** Laura Reche-Olmedo, Laura Torres-Collado, Laura María Compañ-Gabucio, Manuela Garcia-de-la-Hera

**Affiliations:** 1 Unidad de Epidemiología de la Nutrición, Departamento de Salud Pública, Historia de la Ciencia y Ginecología, Universidad Miguel Hernández (UMH), 03550 Alicante, Spain; laura.reche@alu.umh.es (L.R.-O.); l.torres@umh.es (L.T.-C.); manoli@umh.es (M.G.-d.-l.-H.); 2 Instituto de Investigación Sanitaria y Biomédica de Alicante, ISABIAL, 03010 Alicante, Spain; 3 CIBER Epidemiología y Salud Pública (CIBERESP), Instituto de Salud Carlos III, 28034 Madrid, Spain

**Keywords:** occupational therapy, autism, picky eating, neurodevelopmental disorders, scoping review, nutrition

## Abstract

Food selectivity is common in children with autism spectrum disorder (ASD). It can be defined as the unwillingness to eat common or new foods, resulting in a lack of variety in the diet or limited food consumption for multiple reasons, such as inflexibility or sensory alterations. We conducted a peer scoping review to describe the interventions that are carried out from occupational therapy (OT) in children with ASD with food selectivity. Two authors independently searched the databases PubMed, Scopus, Web of Science, and EMBASE, as well as the OT journals indexed in Journal Citation Reports. Articles exploring OT interventions in children (≤12 years) with ASD and food selectivity, published in Spanish or English, with experimental design, and with full text available were included. Of the 1445 articles identified, 8 articles met the inclusion criteria. Three main intervention categories were identified: sensory–behavioral, family focused, and other interventions. Most of the interventions from OT were aimed at treating sensory–behavioral aspects. Only three articles described interventions led exclusively by occupational therapists, and the rest were led by a multidisciplinary team. Finally, although these interventions are not exclusive to OT, occupational therapists can participate together with other professionals as an essential component in the treatment of food selectivity in children with ASD.

## 1. Introduction

Autism spectrum disorder (ASD) is a neurodevelopmental disorder characterized by persistent impairments in communication, social interaction in different contexts, and the presence of restrictive and repetitive behavioral patterns that directly affect social, occupational, and daily functioning [1,2]. The prevalence of this disorder, which is more common in boys, has been increasing in recent decades [3], and it currently affects 62/10,000 children worldwide, which translates into 1 child out of 160 [4,5].

ASD is often associated with sensory difficulties, including hyperreactivity or hyporeactivity to sensory stimuli in the environment [6]. More than 80% of people with ASD present impaired sensory processing or modulation [6], which can have a direct impact on their functionality and many aspects of daily life such as playing, social activities, self-care, and learning [7]. Eating can be a particularly difficult activity for children with ASD due to sensitivity to food textures and also to the difficulty to try unfamiliar foods (i.e., food neophobia) [8].

Children with ASD can be very selective in their eating patterns, a condition known as picky eating [9]. Previously published studies have indicated that picky eating is present in 58% to 67% of children with ASD aged between 3 and 18 years [10,11,12,13]. This condition can be present in children with ASD of any age, but it has a higher severity in children aged less than three years [14]. Indeed, picky eating is most common in the first year of life from the time of introducing the first complementary foods (i.e., vegetables and fruits) [15].

Picky eating can be defined as the unwillingness to eat familiar or new foods [16], resulting in an unvaried diet or limited food consumption [9]. Often, children with ASD refuse to eat foods that are mushy, with tough textures, or with a bitter taste, as well as dishes with bits or hidden ingredients [17]. This rejection may be due to sensory issues that children with ASD have toward certain foods [18]. An example of this would be the tactile or taste sensory sensitivity to fibrous and moist foods, such as fruit and vegetables, or the auditory sensory sensitivity to biting into crunchy foods [16,18]. As food selectivity directly affects nutritional intake, it can negatively influence children’s growth and development [19]. Furthermore, in children with ASD, these negative effects may be exacerbated because many follow specialized diets that exclude gluten or dairy [20], leading to a still more highly restricted diet.

Picky eaters may present sensory issues, in addition to behavioral ones, such as performing rituals before meals, and/or cognitive ones, such as inflexibility [15]. Usually, these impairments, especially inflexibility, can lead to problems in the parent–child relationship [21] because picky eaters reject their parents’ efforts to include healthier foods in their diets. In this sense, both the parents of children with ASD and picky eating and the children themselves can experience stress and anxiety [22,23]; the parents because their children do not have a healthy diet, and the children because they face the inclusion of new foods in their diet [24].

Previous studies have been published on interventions on children with ASD and picky eating. Wood et al. [25] conducted an intervention based on a combination of different strategies, such as contingent reinforcement, physical cues, or procedures to introduce new foods gradually, showing an increase in the number and variety of foods consumed. Similar results to those found by Wood et al. were shown in other published articles [26,27,28]. Najdowski et al. [29] used the simultaneous presentation of different foods and the elimination of pureed texture by the use of texture fading, and they observed a decrease in food selectivity. These studies are examples of physical and cognitive interventions that can be carried out by multidisciplinary teams, which may include occupational therapists, dietitians, speech therapists, or psychologists [30]. One of the most widely used occupational therapy (OT) strategies for the treatment of children with ASD and picky eating is sensory integration therapy (SIT) [9]. However, OT intervention in this field often also involves the family in order to help them on managing selective eating behaviors. In this sense, a previous study showed that a parents program based on the application of new approaches to meal preparation and the establishment of strategies to address picky eating helped to increase the number of foods that children found acceptable [31]. One aspect to consider in interventions is the level of functioning of children, which can vary widely between ASD subtypes, as it is a very heterogeneous group with high differences between ASD subtypes and between children with the same type of ASD [32,33]. However, we did not find any articles that have studied the differences in the success of the picky eating intervention in children with ASD in relation to the subtype of ASD.

When picky eating in children with ASD is caused by altered sensory processing, the occupational therapist is one of those responsible for assessing the sensory profile and identifying alternative foods or alternative strategies in order to provide adequate nutrient intake [16]. However, despite the high prevalence of picky eating in children with ASD, evidence on associated OT interventions is very scarce. In fact, there are no previous reviews investigating this issue. Therefore, we believe that this review can provide occupational therapists and other professionals working with children with ASD with an information resource with which to learn more about OT interventions in picky eating. Thus, the aim of this scoping review is to describe the interventions carried out from OT in children with ASD with food selectivity. Specifically, we address the following question: What interventions are carried out from OT in children with ASD with food selectivity?

## 2. Materials and Methods

We performed a peer scoping review following the recommendations of the *Cochrane Handbook* [34] and those of the PRISMA Extension for Scoping Reviews (PRISMA-ScR) [35]. A protocol of this review has not been published and, since this is a scoping review, it has not been registered with Prospero.

### 2.1. Search Strategy

We conducted a literature search on 25 November 2020. Two reviewers independently carried out the search in four databases: PubMed, Scopus, Web of Science, and EMBASE. In addition, a manual search was carried out in different OT journals indexed in Journal Citation Reports (JCR): *American Journal of Occupational Therapy*; *Journal of Occupation Rehabilitation*; *Physical and Occupation Therapy in Pediatrics*; *Occupations, Participation and Health*; *Scandinavian Journal of Occupational Therapy*; *Australian Occupational Therapy Journal; British Occupational Therapy*; *Canadian Journal of Occupational Therapy*; *Occupational Therapy Intervention*; and *Hong Kong Journal of Occupational Therapy*. We used the same search strategy and keywords in the different databases and scientific journals and used a combination of two searches: (1) (“food selectivity” OR picky) AND (autism OR autistic OR ASD OR Asperger OR Rett OR disintegrative OR pervasive) and (2) (“food selectivity” OR picky) AND (autism OR autistic OR autistic OR ASD OR Asperger OR Rett OR disintegrative OR pervasive) AND “occupational therapy” (Table S1).

### 2.2. Inclusion Criteria

In this review, we included all studies that met the following inclusion criteria: (1) articles whose study population consisted of children (≤12 years old) with Asperger’s syndrome (ASD), Rett syndrome, disintegrative disorder, classic autistic disorder, and pervasive developmental disorder); (2) articles evaluating or exploring OT intervention in food selectivity (picky eating, fussy eating); (3) articles published in English or Spanish; (4) articles whose study design was experimental: randomized or non-randomized studies, pilot studies, case studies, quasi-experimental studies, or exploratory studies; (5) articles with full text available.

We did not use filters by type of study or time during the literature search in any of the journals and databases consulted.

### 2.3. Study Selection and Data Extraction

All titles of articles found in the searches of databases and journals used within this review were downloaded onto a Microsoft Excel spreadsheet. From this excel database, the studies were screened and selected in four steps: elimination of duplicates, screening by title, screening by abstract, and screening by full text. All screening was performed independently by two researchers (L.R.O., L.T.C.). All articles that did not meet the above inclusion criteria were excluded. When there were discrepancies about whether or not to include an article, this was resolved with the help of a third researcher (M.G.H.).

Before data extraction, we developed two tables following the *Cochrane Handbook* recommendations [36] to guide and facilitate the data extraction process, as well as to minimize the possible subjectivity of the researchers. One table contains the main characteristics of the included studies as follows: author, year of publication, country, study design, study sample and population, evaluation, main results, and main limitations of the study. The other table contains the characteristics of the interventions carried out in the included studies: author, year of publication, country, type of food selectivity, OT intervention/comparator, intervention duration, number of sessions, measuring instruments, and the role of the occupational therapists. Two researchers (L.R.O. and L.T.C.) conducted the data extraction independently.

### 2.4. Quality Assessment

The quality of the included studies was not assessed, as this is not a mandatory requirement of scoping reviews [35,37,38,39,40]. However, we showed in the tables the main limitations reported in the included articles to make readers aware of these characteristics. Thus, readers will be able to assess more critically the results presented in each study and in this scoping review. The main limitations reported by authors in each included study are also discussed in the Results Section.

## 3. Results

**Figure 1:**
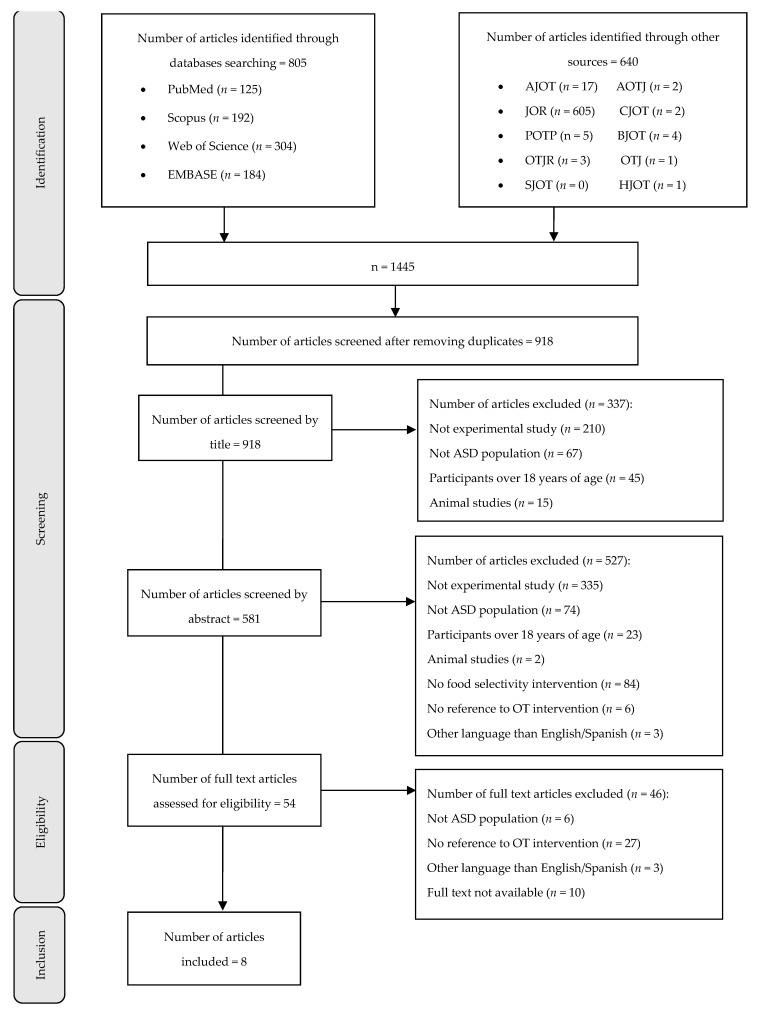
Flowchart of the study selection process: AJOT, *American Journal of Occupational Therapy*; AOTJ, *Australian Occupational Therapy Journal*; BJOT, *British Journal of Occupational Therapy*; CJOT, *Canadian Journal of Occupational Therapy*; JOR, *Journal of Occupation Rehabilitation*; HKOT, *Hong Kong Journal of Occupational Therapy*; OTJ, *Journal of Occupational Therapy*; OTJR, *Occupation, Participation, and Health*; POPT, *Physical and Occupation Therapy in Pediatrics*; SJOT, *Scandinavian Journal of Occupational Therapy*.

The search strategies identified a total of 1445 published articles related to OT interventions in picky eating (Figure 1). Following a peer-review process, 54 articles were selected for retrieval and full-text assessment, while 46 articles were excluded, as they did not meet the inclusion criteria. We extracted data from eight articles that fulfilled the criteria for this scoping review. The flowchart of the study selection is shown in Figure 1**.**

### 3.1. Main Characteristics and Limitations of Included Studies

Table 1 summarizes the main characteristics of the studies included in this scoping review. The eight selected studies were conducted in different countries: the USA (*n* = 6), Mexico (*n* = 1), and Japan (*n* = 1). One of the studies included is a randomized clinical trial [41], one is a non-randomized clinical trial [31], one is a pilot study [42], three are case reports [43,44,45], and two are case series [46,47]. All children who participated in the interventions described in the included articles were diagnosed with ASD. The diagnosis of ASD was confirmed by an interdisciplinary team (*n* = 2) [41,47] using different methods such as the Autism Diagnostic Observation Schedule (ADOS) [41,42], ICD-10 checklist [47], DSM-5 checklist [42], interviews with the children’s parents [41] and review of medical records [46]. In three articles, there is no information on where or how the diagnosis of ASD was made [43,44,45].

Overall, intervention studies showed that after interventions, the quantity of food consumed (in grams) increased in children with autism [31,41]. Specifically, in Peterson’s study, this association was only observed in children who received applied behavioral analysis (ABA) therapy [41]. In addition, included studies showed that the interventions increased the number of foods (food variety) accepted by children [31,41,42,43,44,45,46,47], decreased inappropriate mealtime behaviors [41,43,45,46], and reduced challenging attitudes during mealtime [47].

The main limitation reported regarding the articles included in this scoping review was the small sample size (*n* = 4) [42,44,45,47]. In addition, articles indicate results difficult to generalize (*n* = 2) [42,47], did not evaluate the long-term effects of the interventions (*n* = 2) [46,47], did not provide training for parents after the intervention in order to continue the food presentation at home (*n* = 1) [41], food presentation differed during the intervention (*n* = 1) [41], dietary selectivity was difficult to assess (*n* = 1) [31], a lack of a control group [42], and there could be a carry-over effect of continued sessions (*n* = 1) [46]. Finally, one study did not state limitations [43]. 

### 3.2. Interventions Including OT in Picky Eating

Table 2 presents the characteristics of interventions that included OT described in the studies included in this review—namely, type of food selectivity, type of intervention, duration, number of sessions performed, instruments used to measure the effectiveness of the intervention, and the role or activity carried out by the occupational therapist.

#### 3.2.1. Sensory–Behavioral Interventions

Three studies were identified that conducted behavioral interventions for children with ASD [41,43,46]. The main objectives of these interventions were to improve the acceptance or grams of food consumed [41,46], to reduce inappropriate mealtime behavior [41,43,46], to improve mouth cleans [41,43], and to reduce the presence of packing (i.e., holding food in the mouth larger than a grain of rice), gagging, and spitting out of food [43].

One of the interventions was based on a behavioral feeding intervention program developed in 2018 by Seiverling et al. [46]. This program consisted of two different experimental conditions: with and without sensory integration therapy (SIT) before meals. Both participants received SIT before each meal, one of them on all treatment days and the other on alternate days. SIT was carried out in 15 min pre-meal activities using materials such as a trampoline, a therapeutic mat, or a sensory brush. This study was an 8–15-day intervention during which different types of liquids or pureed foods were administered. The results were an increase in the amount of food and drink consumption and a reduction in inappropriate behavior during mealtime in both types of conditions.

Another included study carried out applied behavior analysis (ABA) treatment, and modified sequenced oral–sensory–oral treatment (M-SOS) [41]. This program focused, on the one hand, on inappropriate eating behavior caused by environmental factors, and on the other hand, on the use of a food texture hierarchy to address food selectivity. During the four weeks of intervention, the study showed an increase in food consumption in children who received the ABA treatment.

Finally, the third study, by Sharp et al. (2009) [43], conducted a daytime treatment program using texture reduction. This study aimed to determine the magnitude of change in food volume and texture that the child would tolerate without the need for texture or volume fading. By providing foods with decreased texture or volume, the child was observed to accept all mouthfuls quickly without nausea or expulsion. Specifically, sensory integration was performed as a series of personalized activities, as the majority of the children were sensory seekers (i.e., children who excessively seek sensory input to self-regulate) [48,49]; the activities were selected to provide proprioceptive and vestibular input. The sensory integration activities lasted 15 min and were performed before the specific feeding intervention [43].

In general, these studies were performed by a multidisciplinary team made up of psychologists, feeding therapists, trained therapists, dietitians, and occupational therapists. The occupational therapists participated in the evaluation of participants [43] in the treatment [41,43], and in the activity development and training of children in the implementation of active sensory integration [46]. These interventions were carried out with children aged between three and six years old. The intervention lasted between 2 and 12 weeks, and there were 4 daily sessions approximately, lasting between 20 and 90 min.

#### 3.2.2. Family-Focused Interventions

A total of three studies conducted interventions targeting families. The main objectives of these interventions were to provide parents with basic skills to manage food selectivity and to implement new tools to reduce food selectivity [31,42] and improve the acceptance of food consumed [47]. Most of these interventions, in addition to involving the family, included sensory integration activities.

The first study aimed to increase food preferences through an intervention program for parents based on the person–environment–occupation model [31]. The intervention aimed to provide parents with basic skills to manage food selectivity and factors influencing food preferences and to implement new approaches and strategies to address food selectivity. This study showed a decrease in the amount of unaccepted food after eight weeks of intervention.

The second study was based on a family-focused food intervention called EAT-UP [47], which aimed to address both food selectivity and inappropriate behaviors, resulting in an increase in the level of food acceptance and food diversity. There was also a decrease in the presence of challenging or inappropriate behaviors.

The third study was based on a cognitive–behavioral treatment “BUFFET”, a food flexibility and exposure treatment program developed by Kuschner et al. (2017) [42]. It is based on developing children’s coping skills to deal with anxious situations and thus know how to act flexibly with novel or non-preferred foods. After a 16-week intervention, it was observed that this type of treatment helped to reduce picky eating.

These interventions involved parents with children aged 3 to 12 years old and were carried out exclusively by occupational therapists who were responsible for interviewing parents [31], intervening [47], developing the intervention program [42], and providing parents with information about food and nutrition and counseling them about their concerns [31]. One exception was the study by Kuschner et al. (2017) in which research assistants and doctoral- or masters-level clinicians also formed part of the intervention team [42]. The duration of these interventions ranged from 8 to 20 weeks. The program for parents based on the person–environment–occupation model was carried out through 40 min sessions [31]. The program EAT-UP was carried out during mealtimes or snack times, but the total number of sessions was not stated [47]. The BUFFET treatment lasted 16 weeks and consisted of one weekly 90 min session [42].

#### 3.2.3. Other Interventions

A total of two articles were included in this category, as they did not fit into any of the previous categories.

One of the studies was based on a multicomponent treatment [44], which included different techniques to address food selectivity such as sensory integration, systematic desensitization, behavior modification, positive reinforcement, and escape extinction. The main variables of this study were negative behaviors and interaction with food. The multicomponent treatment was found to increase food consumption and acceptance. The intervention was carried out with children aged eight years old by an occupational therapist. The intervention program lasted 40 weeks and consisted of one weekly session. The duration of the sessions was not stated.

Finally, Whipple et al. [45] developed a program in 2019 to decrease food selectivity related to packing in order to reduce inappropriate behaviors during mealtime and food selectivity. This study is based on the simultaneous presentation of preferred foods alongside non-preferred foods. In this study, inappropriate behavior, expulsion, packing, and meal duration were assessed. By providing non-preferred foods with five dime-sized bites, it was observed that the child increased the number of foods consumed that had previously been rejected. This intervention was carried out with children aged four years old. In this study, the intervention was carried out by trained therapists, speech–language pathologists, and occupational therapists, who were responsible for the participants’ evaluation. The intervention consisted of 3–4 weekly 45 min sessions. The duration of the intervention was not stated.

## 4. Discussion

The aim of this study was to describe the most commonly used OT interventions in children with ASD and food selectivity. We found few studies on food selectivity interventions led by occupational therapists in children. All the included studies in this scoping review have been published in the last five years, which suggests an increasing trend of publications related to this topic of study in recent years.

Sensory–behavioral interventions were the most investigated interventions in the included studies, and they showed improvements in the amount of food consumed and a decrease in inappropriate feeding behaviors. These results are consistent with those obtained by Seiverling et al. [46], who compared a behavioral dietary intervention with and without sensory integration and found improved behavioral feeding in those participants who received sensory integration. In contrast, the results obtained by Peterson et al. [41] showed that children with ASD who were intervened with applied behavior analysis (ABA) had increased their target food consumption in comparison with those children intervened with a modified sequential oral sensory approach (M-SOS), which is a type of sensory–behavioral intervention. Discrepancies between the results of the two studies can be partly explained by the fact that it is not entirely clear whether picky eating is the consequence or the origin of disruptive behaviors in either typically functioning children [50] or children with ASD [10]. Some previously published studies suggest that picky eating can be produced by environmental factors during meals, such as parental stress [23] or anxiety [51] and other parental feeding practices [22,52], but can also be produced by food availability [53]. In addition, picky eating can also be caused by factors related to children such as emotional problems [54], shyness [55], behavioral problems [56], autistic traits [57], a low level of sociability [58], and sensory modulation problems [59,60], i.e., tactile and/or oral defensiveness (oral hypersensitivity, inability to process sensory information from oral input leading to aversion [61]). In this sense, sensory reeducation intervention provides tactile desensitization activities and play to help the child feel more comfortable with different types and texture foods, leading to a gradual increase in food acceptance and, therefore, a decrease in food selectivity [62].

Family-focused interventions were used as frequently as sensory–behavioral interventions in the included studies. Results of family-focused interventions showed an increase in food preferences and a decrease in inappropriate mealtime behaviors. These results are consistent with the results found by Miyajima et al. [31]; according to their study, an intervention based on the person–environment–occupation model focused on parents can reduce picky eating behaviors in children. Similarly, Cosbey et al. [47] showed that interventions with the family through coaching and feedback can increase the level of food acceptance and food diversity. Finally, Kuschner et al. [42] showed that interventions based on the treatment of food flexibility in a multifamily way help to reduce picky eating behaviors. One reason that could explain the positive effects of family interventions in picky eating is joint family meals. Family meals have been related to an increase in family closeness and in the emotional well-being of all family members, which can reduce pickiness [53,63,64]. It should be noted that the relationship between family meals and food selectivity in children may be influenced by socioeconomic status (SES). In particular, children from low-income families tend to eat less fruit and vegetables, as their families are unable to provide them with a varied diet [65,66]. In this type of approach, parents and other caregivers can provide essential assistance to intervention because, generally, they are most familiar with their child‘s behaviors and preferences [67]. Parents are trained to recognize the source of the feeding problem, and therapists show them different coping skills to prevent food refusals and ensure that the child is regularly exposed to a diversity of food in their daily diet [68]. In addition, family meals are a great opportunity to enjoy each other’s company and exchange feelings and family values, increasing children’s sociability, which is a psychological aspect closely related to food selectivity [58]. Children with ASD prefer to avoid social situations because they feel uncomfortable, especially when these situations are in groups [69]. For this reason, increasing their sociability may help them to cope with mealtimes, which often have a strong social aspect. In this sense, a recently published brief report indicates that low social communication of children with ASD may have a negative effect on mealtimes, leading to food selectivity behaviors [70].

Interventions that were used to a lesser extent also showed favorable results regarding food selectivity. Sharp et al. [43] showed that interventions based on decreasing food texture and volume can improve food acceptance and food variety. This improvement may be due to the fact that a smaller bite produces less of an aversive response in the child, and therefore, it is easier to increase the acceptance of that food [43]. On the other hand, Suarez [44] found that intervention that integrates different techniques (sensory integration, escape extinction, or positive reinforcement) can also produce positive results in food selectivity. Thirdly, Whipple et al. [45] also showed positive results after an intervention based on the simultaneous presentation of preferred and non-preferred foods, increasing the intake and consumption of previously rejected foods.

This scoping review has some limitations that may influence the results obtained. Firstly, few articles included in this review were carried out exclusively by occupational therapists. Most of the studies were performed by multidisciplinary teams that included an occupational therapist. However, occupational therapists may have sufficient tools to carry out these interventions, as they receive training in sensory integration during their university studies, as well as training in early childhood care, although the scientific evidence supporting these skills is still scarce. In addition, no inclusion criteria regarding occupational therapists (i.e., qualification or education) were established. Secondly, we discarded those studies that were not in full text, which could lead to the loss of some important articles in this review. Thirdly, the search was carried out using four main databases and OT journals on the treatment and intervention of feeding selectivity in children with ASD, and although these databases are used by most reviews because of their extensive content, it is possible that we may have missed some important articles. Furthermore, we used the search results of the less specific search strategy, and we also did not use filters, which led to a greater screening of articles. Furthermore, we were not able to analyze the results of each intervention differentiating by subtype of ASD, as most articles did not specify the subtype. Fourthly, the studies included in this review have a small sample size (five articles included only one, two, or three children), since most of the articles are care reports and case series, making it difficult to generalize the results. Fifthly, we did not assess the quality of the included articles, which means that some of the articles included in this review may have low methodological quality. However, we listed the main reported limitation of every included article in Table 1. Finally, there is a wide variety of interventions on picky eating, which could be explained by the lack of evidence on the causes of this food selectivity. In fact, in 2018, health professionals stated “no consensus” in the etiology of picky eating [57], making it difficult to select a unique intervention strategy.

However, this scoping review has several strengths. Firstly, it highlights the wide variety of interventions in food selectivity in children with ASD, in which the occupational therapist participates together with other professionals and is an essential component in the treatment of food selectivity in children with ASD. Furthermore, it highlights the following gaps in knowledge that will open doors for future research: (1) there are no studies on OT intervention in children with ASD and picky eating in Europe, and specifically in Spain. In contrast, the majority of included studies were carried out in the USA. This could be partly explained by the fact that, according to the results of a bibliometric study of OT publications, the USA published the largest number of OT articles [71]. Another explanation for this knowledge gap could be the disparities in the access to health services among children with ASD. A recent systematic review indicated that the USA had the highest access to allied health services and, by contrast, northern Europe had the lowest receipt of both OT and speech–language pathology services [72]. It should be noted that all the studies are international, so it may not wholly reflect the reality of the European or Spanish population; (2) most of the included studies have low methodological quality since their study sample is very small.

This review suggests that OT is a relevant discipline for ASD children with food selectivity. The main interventions led by occupational therapists were sensory–behavioral and family-focused interventions, which showed beneficial effects in food selectivity. However, these results should be interpreted with caution as they are mainly based on case studies. The included studies showed that occupational therapists can not only cooperate in the multidisciplinary team but can also lead multicomponent and family-focused interventions in children with ASD and food selectivity. However, most interventions were carried out by multidisciplinary groups of professionals, including speech therapists, psychologists, dieticians, or occupational therapists. From this, we can infer that food selectivity is a multifactorial problem that affects different aspects of the health of children with ASD, and therefore, the best treatment is one that is carried out by a multidisciplinary team, in which the occupational therapist has a role.

Finally, we would like to underline that this scoping review provides knowledge on food selectivity to facilitate OT evidence-based interventions.

## 5. Conclusions

In conclusion, the main interventions from OT in the treatment of picky eating in children with ASD are those aimed at the sensory–behavioral level of the children and those focused on the family. The identified interventions appear to show an improvement in food selectivity, increasing the number of foods consumed and accepted, as well as reducing the number of situations of inappropriate feeding behavior. Finally, although most of the interventions included in this review are not unique to OT practice, they can be useful for OT practitioners when dealing with children aged 12 and under with ASD and food selectivity.

## Figures and Tables

**Table 1:** Characteristics of the studies included in the scoping review.

Author, Country, Year	Design	Sample (*n*), Age	Evaluation	Main Results	Limitations
Seiverling et al., USA, 2018 [46]	Case series	2, 5–6 years	Baseline, pre-, post-int, 8-w f-u	- Increase in the grams of foods and drinks consumed without inappropriate behaviors in both participants after the interventions (*p*-value NS).	Carry-over effect, lack of f-u, lack of information on the development of the treatment.
Peterson et al., USA, 2015 [41]	Parallel RCT	6, 4–6 years	Baseline, pre-, post-int	- Increase in food consumption in children who received ABA therapy, but not for children who received the M-SOS (*p*-value NS).	No continuous training of parents in M-SOS therapy, food presentation not typical for M-SOS, lack of f-u.
Sharp and Jaquess., USA, 2009 [43]	Case report	1, 3 years	Pre-, post-int	- Improvement in the acceptance of all bites and textures, with no expulsions or gagging (*p*-values NS).	NS
Miyajima et al., Japan, 2017 [31]	nRCT	23, 3–6 years	Baseline, pre-, post-int	- Increase in the number of food items consumed by children (*p* < 0.001), in parents’ degree of self-efficacy (*p* < 0.018), and in the number of recommendations conducted by parents (*p* < 0.001) - Decrease in subjective view of children’s dietary imbalance (*p* < 0.001).	Difficulty in assessing dietary selectivity, difficulties for parents to follow dietary recommendations.
Cosbey and Muldoon., Mexico, 2016 [47]	Case series	3, 6–8 years	Pre-, post-int	- Decrease in the child’s challenging mealtime behaviors and increased food acceptance (*p*-value NS).	Low generalizability of the results, small sample size, limited duration of the intervention.
Suarez., USA, 2014 [44]	Case report	1, 8 years	Each w of int.	- Increase in food acceptance (*p*-value NS).	Small sample size, low effectiveness for severe food selectivity, lack of nutritional status assessment.
Whipple et al., USA, 2019 [45]	Case report	1, 4 years	Baseline, pre-int, 4-w f-u	- Decrease in packing and meal duration (*p*-values NS).	Difficulty in eliminating preferred products, small sample size.
Kuschner et al., USA, 2017 [42]	Open pilot trial	11, 8–12 years	Pre-,post-int,4- and 12-w f-u	- High satisfaction with the intervention (*p*-value NS) and high rate of parents reported that the intervention helps to reduce selective feeding (88%).	No control group, small sample size, low generalizability of the results.

ABA; applied behavioral analysis treatment; ASD, autism spectrum disorder; f-u, follow-up; int, intervention; M-SOS, modified sequential oral sensory sequenced treatment; NS, not stated; RCT, randomized controlled trial; nRCT, non-randomized controlled trial; w, weeks.

**Table 2:** Characteristics of the interventions carried out in the studies included in this scoping review.

Author, Country, Year	Eating Problem	Intervention	Interventions Description	Duration (w)	Sessions	Measuring Instruments	Occupational Therapist’s Role
Seiverling et al., USA, 2018 [46]	Food selectivity, especially with liquids or pureed foods	Behavioral dietary intervention without and with SI therapy	CG: Behavioral feeding intervention without SI. Experimenters alternated between presenting a mouthful of food and a drink. IG: Behavioral feeding intervention with SI. CG intervention + SI activities before each meal (proprioceptive and tactile input).	1–2	8–15 sessions. Four daily 20 min sessions.	- IMB assessed through the observation of video recording. - Food intake assessed through the number of foods consumed it is was consumed within 10 s after presentation. - Total intake was assessed through the comparison of pre- and post-food weight.	Evaluation, activity development and training of experimenters in the implementation of sensory integration activities. MT: psychologists, feeding therapists, occupational therapists.
Peterson et al., USA, 2015 [41]	Food selectivity	ABA and M-SOS	CG: M-SOS. A 6-step hierarchy food presentation, which included visual tolerance, interaction, smell, taste, and eating. IG: ABA. A sequential bites presentation of a single target food (such as broccoli) every 30s until the presentation of five bites.	NS	1,5 h session. Number and frequency of sessions NS.	- IMB assessed through the observation of video recording. - Total grams of food consumed assessed through the observation of video recording. - Acceptance assessed through the observation of video recording. - Mouth clean assessed through the observation of video recording.	Checking the integrity of the treatment. MT: Therapists and occupational therapists.
Sharp and Jaquess., USA, 2009 [43]	Food selectivity and food rejection	Day treatment program through texture reduction	16 foods were initially presented at pureed texture using a 2-pea bite size. During sessions, both texture and bite size were gradually increased.	4	4 daily 30- to 45 min meals/sessions. Number of sessions NS.	- Mouth clean assessed through observation. - IMB assessed through observation. - Expulsions assessed through observation. - Gagging assessed through observation.	Assessment and treatment to increase oral motor skills. MT: psychologists, dietitians, and occupational therapists.
Miyajima et al., Japan, 2017 [31]	Food selectivity	Intervention for parents based on the person–environment–occupation model.	Seminars for parents of children with ASD. The intervention helped parents learn about selective eating and, therefore, improve their children’s care.	8	2 sessions and two discussions. One-monthly 40 min sessions.	- Degree of difficulty experienced by parents assessed through a VAS. - Degree of parents’ self-efficacy assessed through the SAPS. - Number of recommendations implemented by parents assessed through a 50 items questionnaire. - Changes in the eating patterns of children with ASD assessed through the number of foods that the children chose to eat (47 items) and the parents’ subjective view of the degree of dietary imbalance (VAS).	Interviewing and counseling parents on how to address concerns. No MT.
Cosbey and Muldoon., Mexico, 2016 [47]	Food selectivity and inappropriate behavior	Family centered food intervention EAT-UP	Phase 1 “coaching intervention”: sessions with coaching and feedback, such as visual supports. Phase 2 “stand-alone intervention”: sessions with only feedback.	20	Number of sessions varied between participants (5–21 sessions). Duration of sessions NS	- Children’s food acceptance and dietary diversity were assessed through the use of a Food Frequency Questionnaire and a 24 h food recall. - Children’s mealtime behaviors assessed through the BAMBI and the BPFAS.	Intervention. No MT.
Suarez., USA, 2014 [44]	Food selectivity	Multicomponent treatment	A combination of sensory integration, systematic desensitization, behavior modification, positive reinforcement, extinction of escape and parent and home education.	40	22 sessions. 1-weekly session. Duration of sessions NS	- Children’s food consumption assessed through a food inventory. - Children’s sensory processing difficulties assessed through the SSP.	Intervention. No MT.
Whipple et al., USA, 2019 [45]	Food selectivity, packing and inappropriate behaviors	Simultaneous presentation	Simultaneous presentation of preferred and non-preferred foods on the spoon.	NS	50 sessions. Three—to four—weekly 45 min sessions.	- Packing frequency assessed through the observation of video recording. - IMB assessed through the observation of video recording. - Expulsions assessed through the observation of video recording. - Meal duration assessed through the observation of video recording.	Oral motor skills assessment. MT: Trained therapists and occupational therapists.
Kuschner et al., USA, 2017 [42]	Food selectivity	BUFFET cognitive–behavioral treatment (food flexibility and exposure treatment program)	Multifamily intervention. Children were helped to develop strategies to act flexibly with new or non-preferred foods.	16	14 sessions. One-weekly 90 min sessions.	- Acceptability of BUFFET assessed through session attendance, and individual session ratings. - Global parent satisfaction assessed through the CSQ-8	Development of the intervention program. MT: Trained research assistants, doctoral- or masters-level clinicians, and occupational therapists.

ABA, applied behavior analysis treatment; ASD, autism spectrum disorder; BAMBI, Brief Autism Mealtime Behavior Inventory; BPFAS, Behavioral Pediatrics Feeding Assessment Scale; CG, control group; CSQ-8, client satisfaction questionnaire; IG, intervention group; IMB, inappropriate mealtime behavior; M-SOS, modified sequential oral sensory sequenced treatment; MT, multidisciplinary team; NS, not stated; SAPS, assessment for parents of children with selective eating; SI, sensory integration; SSP, short sensory profile; VAS, visual analog scale; w, weeks.

## Data Availability

The data presented in this study are available on request from the corresponding author. The data are not publicly available due to confidentiality and ethical reasons.

## Supplementary Materials

The following are available online at https://www.mdpi.com/article/10.3390/children8111024/s1, Table S1: Databases and search strategies.
