# Supplementary material for: The Role of Occupational Therapy in Managing Food Selectivity of Children with Autism Spectrum Disorder: A Scoping Review
Source: Children (Basel). 2021 Nov 7;8(11):1024. doi: 10.3390/children8111024 (PMC8620957; doi:10.3390/children8111024)
Supplement: Supplementary file 1 [file children-08-01024-s001.zip › children-1436294-supplementary.pdf]

Table S1. Databases and search strategies.

Database/journal	Search strategies	Results
**Embase**		
1	('food selectivity'/exp OR 'food selectivity' OR picky) AND ('autism'/exp OR autism OR autistic OR 'asd'/exp OR asd OR asperger OR rett OR disintegrative OR pervasive)	184
2	('food selectivity'/exp OR 'food selectivity' OR picky) AND ('autism'/exp OR autism OR autistic OR 'asd'/exp OR asd OR asperger OR rett OR disintegrative OR pervasive) AND 'occupational therapy'	13
3	#1 OR #2	184
**PubMed**		
1	("food selectivity" OR picky) AND (autism OR autistic OR ASD OR asperger OR rett OR disintegrative OR pervasive)	125
2	("food selectivity" OR picky) AND (autism OR autistic OR ASD OR asperger OR rett OR disintegrative OR pervasive) AND "occupational therapy"	7
3	#1 OR #2	125
**Scopus**		
1	(“food selectivity” OR picky) AND (autism OR autistic OR asd OR asperger OR rett OR disintegrative OR pervasive)	1,098
2	TITLE-ABS-KEY ((“food selectivity” OR picky) AND (autism OR autistic OR asd OR asperger OR rett OR disintegrative OR pervasive))	192
3	TITLE-ABS-KEY (“food selectivity” OR picky) AND (autism OR autistic OR asd OR asperger OR rett OR disintegrative OR pervasive) AND “occupational therapy”	2
4	TITLE-ABS-KEY ((“food selectivity” OR picky) AND (autism OR autistic OR asd OR asperger OR rett OR disintegrative OR pervasive)) AND TITLE-ABS-KEY (“food selectivity” OR picky) AND (autism OR autistic OR asd OR asperger OR rett OR disintegrative OR pervasive) AND “occupational therapy”	192
**Web of science**		
1	(("food selectivity" OR picky) AND (autism OR autistic OR ASD OR asperger OR rett OR disintegrative OR pervasive))	304
2	(("food selectivity" OR picky) AND (autism OR autistic OR ASD OR asperger OR rett OR disintegrative OR pervasive) AND "occupational therapy")	9
3	#2 OR #1	304
**American Journal of Occupational Therapy**		
1	("food selectivity" OR picky) AND (autism OR autistic OR ASD OR asperger OR rett OR disintegrative OR pervasive)	17
2	("food selectivity" OR picky) AND (autism OR autistic OR ASD OR asperger OR rett OR disintegrative OR pervasive) AND "occupational therapy	6
**Journal of Occupation Rehabilitation**		
1	("food selectivity" OR picky) AND (autism OR autistic OR ASD OR asperger OR rett OR disintegrative OR pervasive)	605
2	("food selectivity" OR picky) AND (autism OR autistic OR ASD OR asperger OR rett OR disintegrative OR pervasive) AND "occupational therapy"	95
**Physical & Occupation Therapy in Pediatrics**		

1	[All: asd] OR [All: autism] OR [All: autistic] OR [All: asperger] OR [All: rett] OR [All: pervasive] OR [All: disintegrative] AND [in Journal: Physical & Occupational Therapy In Pediatrics]	5
2	[[All: "food selectivity"] OR [All: picky]] AND [[All: autism] OR [All: autistic] OR [All: asd] OR [All: asperger] OR [All: rett] OR [All: disintegrative] OR [All: pervasive]] AND [All: "occupational therapy"] AND [in Journal: Physical & Occupational Therapy In Pediatrics]	4
**Occupation, Participation & Health**		
1	[[All "food selectivity"] OR [All picky]] AND [[All autism] OR [All autistic] OR [All asd] OR [All asperger] OR [All rett] OR [All disintegrative] OR [All pervasive]]	3
2	[[All "food selectivity"] OR [All picky]] AND [[All autism] OR [All autistic] OR [All asd] OR [All asperger] OR [All rett] OR [All disintegrative] OR [All pervasive]] AND [All "occupational therapy"]	3
**Scandinavian Occupational Therapy**		
1	[[All: autism] OR [All: autistic] OR [All: asd] OR [All: asperger] OR [All: rett] OR [All: disintegrative] OR [All: pervasive]] AND [All: feeding] AND [in Journal: Scandinavian Journal of Occupational Therapy]	0
2	[[All: "food selectivity"] OR [All: picky]] AND [[All: autism] OR [All: autistic] OR [All: asd] OR [All: asperger] OR [All: rett] OR [All: disintegrative] OR [All: pervasive]] AND [All: "occupational therapy"] AND [in Journal: Scandinavian Journal of Occupational Therapy]	0
**Australian Occupational Therapy Journal**		
1	"("food selectivity" OR picky) AND (autism Oautistic OR ASD OR asperger OR rett OR disintegrative OR pervasive)" anywhere published in "Australian Occupational Therapy Journal"	2
2	"("food selectivity" OR picky) AND (autism OR autistic OR ASD OR asperger OR rett OR disintegrative OR pervasive) AND "occupational therapy"" anywhere published in "Australian Occupational Therapy Journal"	2
**Canadian Journal of Occupational Therapy**		
1	[[All "food selectivity"] OR [All picky]] AND [[All autism] OR [All autistic] OR [All asd] OR [All asperger] OR [All rett] OR [All disintegrative] OR [All pervasive]] within Canadian Journal of Occupational Therapy	2
2	[[All "food selectivity"] OR [All picky]] AND [[All autism] OR [All autistic] OR [All asd] OR [All asperger] OR [All rett] OR [All disintegrative] OR [All pervasive]] AND [All "occupational therapy"] within Canadian Journal of Occupational Therapy	1
**British Journal of Occupational Therapy**		
1	[[All "food selectivity"] OR [All picky]] AND [[All autism] OR [All autistic] OR [All asd] OR [All asperger] OR [All rett] OR [All disintegrative] OR [All pervasive]] within British journal of occupational therapy	4
2	[[All "food selectivity"] OR [All picky]] AND [[All autism] OR [All autistic] OR [All asd] OR [All asperger] OR [All rett] OR [All disintegrative] OR [All pervasive]] AND [All "occupational therapy"] within British journal of occupational therapy	4

**Journal of Occupational Therapy**		
1	"("food selectivity" OR picky) AND (autism OR autistic OR ASD OR asperger OR rett OR disintegrative OR pervasive)" anywhere published in "Occupational Therapy International"	1
2	"("food selectivity" OR picky) AND (autism OR autistic OR ASD OR asperger OR rett OR disintegrative OR pervasive) AND "occupational therapy"" anywhere published in "Occupational Therapy International"	1
**Hong Kong Journal of Occupational Therapy**		
1	[[All "food selectivity"] OR [All picky]] AND [[All autism] OR [All autistic] OR [All asd] OR [All asperger] OR [All rett] OR [All disintegrative] OR [All pervasive]] within Hong kong Journal of Occupational therapy	1
2	[[All "food selectivity"] OR [All picky]] AND [[All autism] OR [All autistic] OR [All asd] OR [All asperger] OR [All rett] OR [All disintegrative] OR [All pervasive]] AND [All "occupational therapy"] within Hong kong Journal of Occupational therapy	1
